# A case of chronic expanding hematoma mimicking a cystic pancreatic tumor

**DOI:** 10.1186/s40792-024-01957-z

**Published:** 2024-06-26

**Authors:** Asuna Sakamoto, Yasuhisa Ando, Dongping Feng, Mina Nagao, Hiroyuki Matsukawa, Bunpei Nishiura, Akihiro Kondo, Hironobu Suto, Eisuke Asano, Takayoshi Kishino, Minoru Oshima, Kensuke Kumamoto, Keiichi Okano

**Affiliations:** Department of Gastroenterological Surgery, Faculty of Medicine, Kagawa University, 1750-1, Ikenobe, Miki-Cho, Kita-Gun, Kagawa, 761-0793 Japan

**Keywords:** Chronic expanding hematoma, Hand-assisted laparoscopic surgery, Pancreatic cyst

## Abstract

**Background:**

A chronic expanding hematoma is an uncommon entity described as an organized blood collection that increases in size after the initial hemorrhagic event without histological neoplastic features. The standard treatment is complete resection. To our knowledge, this is the first report of a chronic expanding hematoma mimicking a pancreatic cystic tumor that has been successfully resected utilizing a laparoscopic approach.

**Case presentation:**

We report the case of a 32-year-old man with a 10-cm chronic expanding hematoma that was preoperatively diagnosed as a cystic pancreatic tumor. Dynamic computed tomography revealed a cyst at the inferior part of the uncinate process of the pancreas without contrast enhancement. His blood biochemical data were within normal limits. The operation initially utilized a laparoscopic approach; however, the procedure was converted to hand-assisted laparoscopic surgery due to capsule adherence to surrounding organs and finally, enucleation of the tumor was performed. Pathological findings revealed a chronic expanding hematoma in the retroperitoneal space.

**Conclusion:**

Chronic expanding hematoma in the retroperitoneal space is so rare and sometimes adheres to the surrounding tissue. It is difficult to distinguish hematoma attaching pancreas and pancreatic cyst preoperatively. In rare cases such as this, hand-assisted laparoscopic surgery is a feasible, less invasive procedure for facilitating complete resection and preventing recurrence.

## Background

Generally, hematomas can develop due to trauma, surgery, or bleeding disorders, and are naturally reabsorbed without causing problems. However, a chronic expanding hematoma (CEH) fails to resorb over time and exhibits chronic growth [1]. The standard treatment for CEH is complete resection, including capsule resection.

Herein, we report a case of a large CEH arising from the retroperitoneal space that was preoperatively diagnosed as a pancreatic cystic tumor and resected using hand-assisted laparoscopic surgery (HALS). The surgery initially employed a laparoscopic approach but was converted to HALS because the hematoma capsule firmly adhered to the pancreas and duodenum.

## Case presentation

A 32-year-old man was referred to our hospital for a pancreatic cyst detected on abdominal ultrasound during a medical checkup. The patient had no situation related to bleeding diathesis, such as the history of trauma, surgery, anticoagulant drugs, and antiplatelet drugs. His blood biochemical data were within normal limits with a white blood cell count at 5430/µL, red blood cell count of 543 × 10^4^/µL, platelet count at 22.7 × 10^4^/µL, hemoglobin (Hb) at 16.0 g/dL, aspartate aminotransferase at 25 U/L, alanine aminotransferase at 26 U/L, total bilirubin at 0.8 mg/dL, albumin at 4.5 g/dL, C-reactive protein at 0.36 mg/dL, prothrombin time at 115%, activated partial thrombin time at 31.0 s, carcinoembryonic antigen at 0.9 ng/ml (reference range, 0–4 ng/mL), and carbohydrate antigen 19–9 at 2 U/mL (reference range, 0–37 U/mL).

**Fig. 1:**
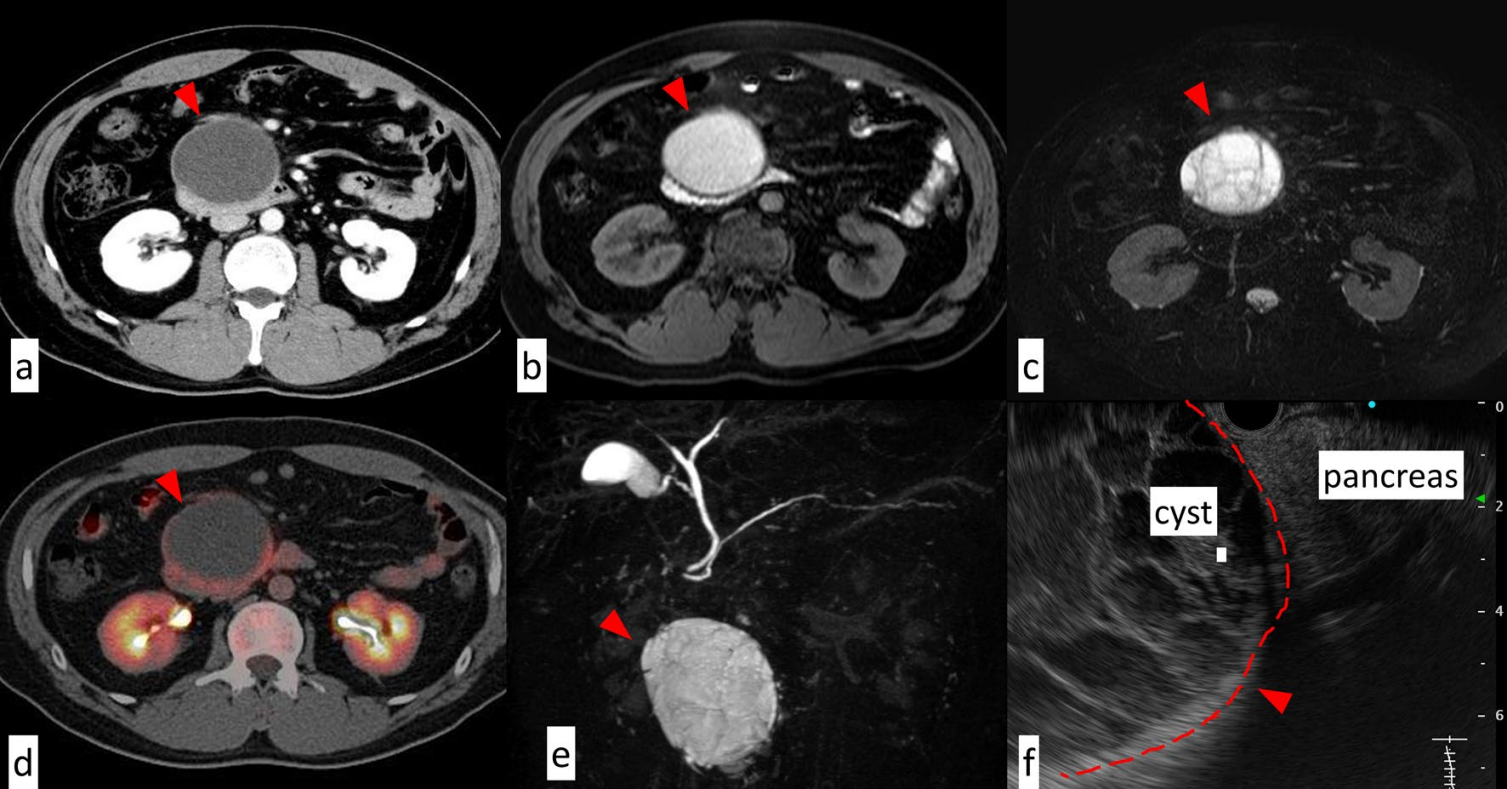
Images. Dynamic computed tomography (CT) showed the cyst, 10 cm in diameter, at the uncinate process of the pancreas without contrast enhancement (**a**). In magnetic resonance imaging (MRI), the cyst had homogeneous high signal intensity on T1-weighted images (**b**) and inhomogeneous signal intensity on T2-weighted images (**c**). No abnormal fluorodeoxyglucose (FDG) accumulation was observed on the FDG-positron emission tomography (PET) images (**d**). In MRCP, the cyst had no communication with pancreatic duct (**e**). Endoscopic ultrasonography (EUS) showed the cyst compressing pancreas, not arising from it (**f**)

Dynamic computed tomography (CT) revealed a cyst, 10 cm in diameter, at the inferior part of the uncinate process of the pancreas. No abnormal fluorodeoxyglucose (FDG) accumulation was observed on the FDG-positron emission tomography (PET) images. Magnetic resonance imaging (MRI) revealed that the cyst had homogeneous high signal intensity on T1-weighted images and inhomogeneous signal intensity on T2-weighted images, known as the mosaic sign. The cyst was not in contact with the main pancreatic duct. Endoscopic ultrasonography (EUS) showed no abnormal vascular flow in the cyst and it had no communication with the ductal system and no malignant features (Fig. 1). Therefore, we diagnosed it as a benign or premalignant lesion, possibly a serous cystic neoplasm (SCN), solid pseudopapillary neoplasm (SPN), or mucinous cystic neoplasm (MCN). Consequently, we decided to enucleate the pancreatic cyst completely for pathological diagnosis instead of pancreaticoduodenectomy.

**Fig. 2:**
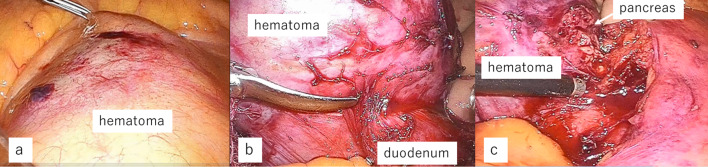
Operation findings. The cyst existed in the retroperitoneal space and firmly adhered to the surrounding tissue (**a**). It adhered to the duodenum, so the serosa was resected with the capsule and directly sutured for repair (**b**). It also adhered to pancreas, so small part of pancreatic tissue was resected for complete resection of the capsule (**c**)

The operation initially employed a laparoscopic approach with five ports. On inspection, the cyst appeared to be firmly adhered to the duodenum and pancreas within the retroperitoneal space (Fig. 2). Therefore, we made a 7-cm incision in the upper abdomen and converted the procedure to hand-assisted laparoscopic surgery (HALS). Palpating the cyst directly enabled enucleation of the tumor along the capsule without damaging the pancreas. The duodenum was so strictly adhered that a portion of the serosa was resected with the capsule and directly sutured for repair.

**Fig. 3:**
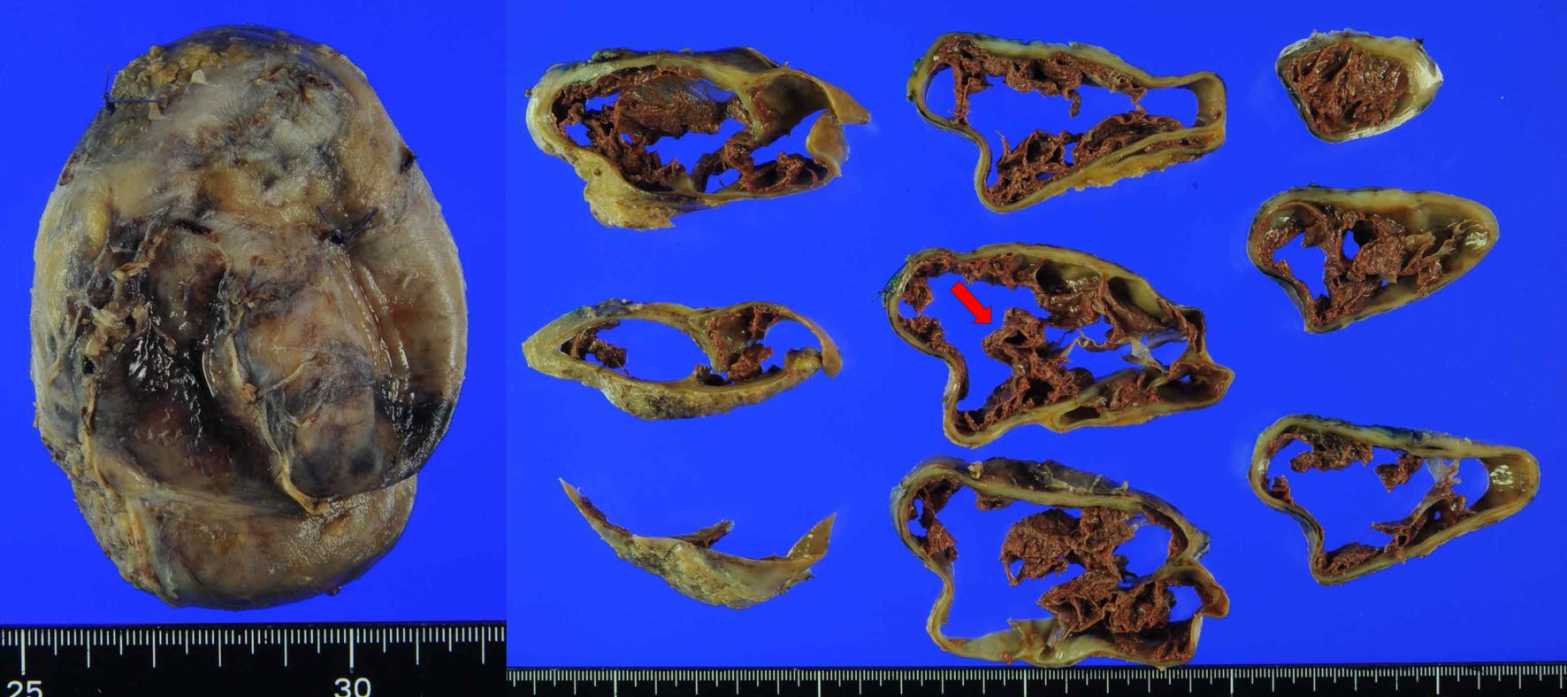
Macroscopic findings. The cyst was 10 cm in diameter and encased in a thick capsule. It had a mosaic-like appearance with septum-like content (arrow)

**Fig. 4:**
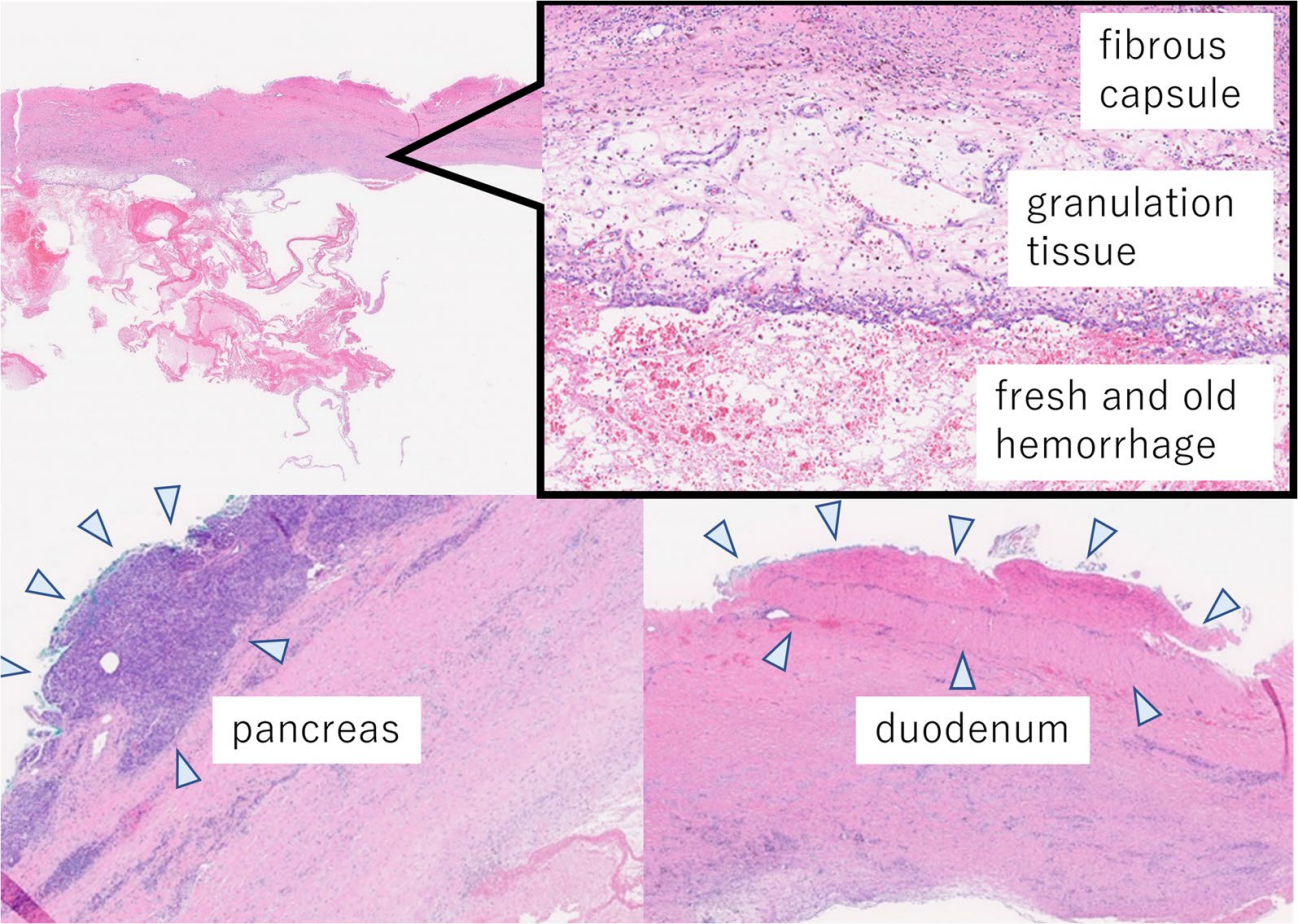
Pathology. The cyst consisted of a central mass of fresh and old hemorrhages, a wall of granulation tissue, and a dense fibrous capsule without neoplastic changes. It was firmly adhered to the pancreas and duodenum (arrowheads)

The cyst was 10 cm in diameter and encased in a thick capsule. It had a mosaic-like appearance with septum-like content and a dark brown fluid (Fig. 3). Pathologically, the cyst consisted of a central mass of fresh and old hemorrhages, a wall of granulation tissue, and a dense fibrous capsule without neoplastic changes. Although it was firmly adhered to the pancreas and duodenum, it did not originate from them (Fig. 4). The pathological diagnosis was chronic expanding hematoma (CEH) in the retroperitoneal space.

The postoperative course was uneventful, with no recurrence after 22 months.

## Discussion

A CEH is an uncommon entity described as an organized blood collection that increases in size for more than a month after the initial hemorrhagic event without histological neoplastic features. These are distinct from general soft-tissue hematomas, which form acutely in the presence of bleeding diatheses, anticoagulant therapy, surgery, or trauma, resolve spontaneously, and gradually decrease in size. Pathologically, CEH is characterized by a central mass of blood, a wall of granulation tissue, and dense peripheral fibrous tissue. Its pathogenic mechanism is not fully understood. However, it is assumed to be caused by an inflammatory response inside the cyst. The fibrin matrix and cellular breakdown products of leukocytes, erythrocytes, hemoglobin, and platelets activate the inflammatory process resulting into a well-defined fibrous cavity [2]. These factors increase vascular wall permeability and bleeding from dilated capillaries in the granulation tissue, resulting in the subsequent growth of the hematoma. CEH can develop at any location in the body, such as the subcutaneous tissue and muscles of the arm or legs, brain, and thorax, but rarely in the abdomen. In a review of 204 CEH cases by Syuto et al., only 10 cases located in the abdomen. Among these, only 7 cases were found in the retroperitoneal space, which located around kidney or iliac fossa [3]. In the pleural cavity, respiratory movements and constant coughing can cause the growth of hematomas. In our case, the constant movement of the duodenum and aortic pumping could have caused hematoma growth [4]. Most CEHs start out as very small traumatic hematomas, though, as in the present case, detecting the initial hematoma is difficult, which means suspecting CEH from medical history would be almost impossible.

However, the way to diagnose with CEH in image inspections has not been established. CEH often exhibits a mosaic sign in T2-weighted MRI, which represents fresh and old blood, indicating repeated bleeding in the cyst [5], like in this case. This is typical of CEH, although hemorrhage from tumor lesions cannot be completely ruled out. As for PET-CT, the FDG uptake seen only in peripheral rim of the mass, reflecting the inflammatory cell infiltration and granulation tissue in the wall, is said to be characteristic in CEH [6], however, it was not detected in our case. Besides, CEH resembles any tumor depending on the location. In this case, the hematoma located at the pancreatic head and mimicked pancreatic cyst. Searching in PubMed with the keyword of ‘chronic expanding hematoma’ and ‘retroperitoneal’, we found 9 cases of CEH in retroperitoneal space, all of them were Japanese reports. Most of them were located at comparably wide space, such as around kidney or iliopsoas muscle, and none of them contacted pancreas. Hisa et al. reported MCN accompanied by a huge mural organized hematoma with internal capillary vessels and hemorrhaging, which seems to be pathologically and morphologically similar to CEH [7]. So, distinguishing CEH compressing the pancreas from MCN with huge cyst seems to be difficult. In retrospect, the EUS in this case indicated that the cyst was compressing the pancreas without originating from it, which is uncharacteristic of pancreatic cysts. The EUS findings might be useful for estimating the origin of the cystic lesion and it is important for surgical strategy to perform neither too much nor too little resection.

Treatment for CEH involves the complete removal of the fibrous capsule, which is responsible for bleeding inside the hematoma. Recurrence after incomplete resection has been previously reported [8]. Moreover, continuous long-term endothelial stimulation in CEH can contribute to neoplastic transformation. A previous report has noted that angiosarcomas can arise from chronic expanding hematomas at the periphery of the pseudocapsule [9]. Weiss et al. reported that hematomas were associated with approximately 5% of malignant fibrous histiocytomas [10]. Besides, it is impossible to diagnose as hematoma preoperatively and we performed the operation suspecting a benign or premalignant tumor such as SPN or MCN. It means damaging the cyst wall causes the risk of peritoneal dissemination and mucus leakage. Consequently, a complete resection is required. However, it is sometimes difficult because of abundant vascularization, leading to massive bleeding and fibrous adhesions to neighboring organs. There are reports of concurrent resections of the lung [4] or bile duct. Conversely, in some cases, resection of entire pseudocapsule was difficult due to adhesions to the aorta [3] or chest wall [11]. In our case, the cyst was sizeable and adhered to the pancreas and duodenum, however, it didn’t touch them so broadly and had distance with the pancreatic duct, so we decided to perform enucleation, instead of pancreaticoduodenectomy.

In such case, adding a hand-assisted port enabled us to precisely recognize the capsule margins and secure the visual field of the operation through cautious compression of the hematoma, which enables oncologically safe and minimally invasive operation.

## Conclusion

CEH in the retroperitoneal space is so rare that diagnostic and surgical methods have not yet been established for it. Complete CEH resection using a laparoscopic approach is occasionally difficult because of capsule adherence to neighboring organs. The HALS procedure is a useful surgical method to employ on large cysts with adherence to the pancreas and duodenum allowing for the complete resection of a large CEH mimicking a pancreatic cyst.

## Data Availability

The data supporting the findings of this study are available as supplementary material.

## Abbreviations

CEH: Chronic expanding hematoma
HALS: Hand-assisted laparoscopic surgery
CT: Computed tomography
PET: Positron emission tomography
MRI: Magnetic resonance imaging
EUS: Endoscopic ultrasonography
SCN: Serous cystic neoplasm
SPN: Solid pseudopapillary neoplasm
MCN: Mucinous cystic neoplasm
